# Microcirculation improvement after short-term infusion of vasopressin in septic shock is dependent on noradrenaline

**DOI:** 10.6061/clinics/2017(12)06

**Published:** 2017-12

**Authors:** Ana Paula Metran Nascente, Flávio Geraldo Rezende Freitas, Jan Bakker, Antônio Tonete Bafi, Renata Teixeira Ladeira, Luciano Cesar Pontes Azevedo, Alexandre Lima, Flavia Ribeiro Machado

**Affiliations:** I Departamento de Anestesiologia, Dor e Terapia Intensiva, Universidade Federal de Sao Paulo, Sao Paulo, SP, BR; II Department of Intensive Care Adults, Erasmus MC - University Hospital Rotterdam, the Netherlands; III Division of Pulmonary, Allergy and Critical Care, Department of Medicine. College of Physicians & Surgeons of Columbia, University of New York, USA

**Keywords:** Septic Shock, Vasopressin, Microcirculation, Vasopressors, Hemodynamic

## Abstract

**OBJECTIVES:**

To assess the impact of vasopressin on the microcirculation and to develop a predictive model to estimate the probability of microcirculatory recruitment in patients with septic shock.

**METHODS:**

This prospective interventional study included patients with septic shock receiving noradrenaline for less than 48 hours. We infused vasopressin at 0.04 U/min for one hour. Hemodynamic measurements, including sidestream dark-field imaging, were obtained immediately before vasopressin infusion, 1 hour after vasopressin infusion and 1 hour after vasopressin withdrawal. We defined patients with more than a 10% increase in total vascular density and perfused vascular density as responders. ClinicalTrials.gov: NCT02053675.

**RESULTS:**

Eighteen patients were included, and nine (50%) showed improved microcirculation after infusion of vasopressin. The noradrenaline dose was significantly reduced after vasopressin (*p*=0.001) and was higher both at baseline and during vasopressin infusion in the responders than in the non-responders. The strongest predictor for a favorable microcirculatory response was the dose of noradrenaline at baseline (OR=4.5; 95% CI: 1.2-17.0; *p*=0.027). For patients using a noradrenaline dose higher than 0.38 mcg/kg/min, the probability that microcirculatory perfusion would be improved with vasopressin was 53% (sensitivity 78%, specificity 77%).

**CONCLUSIONS:**

In patients with septic shock for no longer than 48 h, administration of vasopressin is likely to result in an improvement in microcirculation when the baseline noradrenaline dose is higher than 0.38 mcg/kg/min.

## INTRODUCTION

In patients with septic shock, noradrenaline is usually administered to achieve an adequate mean arterial pressure (MAP) to maintain sufficient organ perfusion. However, adrenergic receptors are hyposensitive during the advanced stages of septic shock 1,2, and the use of a high noradrenaline dose in these circumstances is associated with adverse events 3-6. Excessive use of adrenergic drugs is associated with not only undesirable hemodynamic effects but also enhanced coagulation, reduced innate and adaptive immunity and increased bacterial replication and virulence 7. Thus, the rationale for developing strategies aiming to sparingly use catecholamines in critically ill patients is strong.

Vasopressin has been used as an adjunct to noradrenaline for severe hypotension. A previous meta-analysis of randomized trials suggested improved survival in patients with septic shock who received vasopressin 8-10, although a recent study failed to show improvement in renal dysfunction 11. In addition, several studies have demonstrated that adding vasopressin to a noradrenaline infusion decreases catecholamine requirements 12-17.

Microcirculatory alterations are a hallmark of sepsis, are associated with outcomes 18-20 and are stronger predictors than global hemodynamic variables 21. The effects of vasopressin on the microcirculation have not been adequately studied. The strong vasoconstrictive action, which might hamper microcirculatory flow, of vasopressin is a focus of concern 22. However, the effects of vasopressin on V1 receptors in combination with the reduced dose of adrenergic vasopressors could potentially lead to improved perfusion at the microcirculatory level despite potential negative effects on macrocirculatory parameters such as cardiac output 23-26.

Based on these observations, we carried out a prospective study to evaluate the effects of vasopressin on microcirculatory parameters. In addition, we assessed potential predictive factors related to microcirculation recruitment by vasopressin in septic patients using noradrenaline to sustain MAP.

## MATERIALS AND METHODS

### Study population

This prospective study was conducted in a 35-bed intensive care unit of a teaching hospital. Between June 2010 and August 2012, we included patients with septic shock who received adrenergic vasopressors for less than 48 hours and were monitored with an arterial catheter and a pulmonary artery catheter because we required the monitoring of the cardiac index (CI). According to the unit protocol, in the absence of contraindications, patients with septic shock using noradrenaline above 0.3 mcg/kg/min during the first 24 hours of shock were monitored with a pulmonary artery catheter. Sepsis was defined according to the Society of Critical Care Medicine-American College of Chest Physicians Consensus Conference 27. Septic shock was defined as fluid-refractory hypotension requiring vasopressors with no requirement of elevated lactate levels because the study was conducted before the new definition of septic shock 28. Exclusion criteria included the following: use of vasopressin; acute coronary disease; suspected or confirmed acute mesenteric ischemia; severe hyponatremia (Na+ <130 mmol/L); Raynaud’s phenomenon; systemic sclerosis; pregnancy; or a technical difficulty preventing sublingual video microscopy. The study was conducted according to the Helsinki Declaration, which was revised in 1983, and according to the Resolution 196/96 of the Conselho Nacional de Saude. The Research and Ethics Committee of the institution approved study number 2081/08, and all patients or their legal representatives provided informed consent. ClinicalTrials.gov: NCT02053675.

### Measurements

The demographic and sepsis characteristics and the severity scores from the APACHE II and SOFA were recorded. Hemodynamic measurements included semi-continuous thermodilution CI (Vigilance, Edwards LifeSciences, Irvine, CA, USA), heart rate (HR), MAP, central venous pressure (CVP), pulmonary arterial pressure (PAP), and pulmonary arterial occlusion pressure (PAOP). Ventilator settings were recorded and arterial and mixed venous blood were collected for blood gases analysis, oxygen venous saturation (SvO_2_) and serum lactate.

We assessed sublingual microcirculation using sidestream dark-field (SDF) imaging (Microscan, MicroVision Medical, Amsterdam, Netherlands). To ensure image quality, a skilled physician using the recommended techniques obtained all of the images 29. Three high-quality steady images of at least 20 seconds on both sides of the tongue were obtained while avoiding pressure artifacts. All images were captured using a portable computer and an analog/digital video converter. Microcirculatory parameters, including the microcirculatory flow index (MFI), total vascular density (TVD), proportion of perfused vessels (PPV), perfused vascular density (PVD) and heterogeneity index (HI) 29, were analyzed using AVA 3.0® software (MicroVision Medical, Amsterdam, Netherlands). We obtained only images that were related to vessels with diameters less than 20 µm. We assigned a random number to each image, and the investigator (A.P.M.N.) who analyzed the images was blinded to the patients and details associated with the images.

### Study protocol

Immediately before vasopressin infusion, each patient was evaluated for adequate intravascular volume as evidenced by pulse pressure variation assessment (DX 2020, Dixtal, São Paulo, Brazil) after adequate continuous sedation to control spontaneous ventilatory efforts. Patients with a pulse pressure variation >13% received repeated Ringer’s lactate challenges until the pulse pressure variation was below this value. Patients with pulse pressure variations that could not be measured received fluid challenges until no increase in cardiac output greater than 10% was evident. Vasopressors were used to maintain MAP above 65 mmHg. The oxygen inspiratory fraction was adjusted to maintain peripheral oxygen saturation above 92%.

Thirty minutes after the initial stabilization, we obtained the baseline hemodynamic, respiratory, and sublingual microcirculatory measurements (T0). After the baseline measurements, vasopressin was administered at a fixed dose of 0.04 U/min. One hour after vasopressin infusion, we collected the data from the same variables (T1). Vasopressin was stopped, and new measurements were recorded after 1 hour (T2). If clinically required, the vasopressor infusion was adjusted during the study period to maintain a target MAP level >65 mmHg. If the patients received dobutamine, the doses were kept constant during the study procedure. Patients were excluded from the study if there was a clinical indication of tapering of the ventilator parameters or additional sedation during the intended study period.

### Statistical analysis

Data are expressed as the mean±standard deviation, median [interquartile range], or frequency (n, %), as appropriate. The time points of the study were defined as T0 (baseline before vasopressin infusion), T1 (one hour after vasopressin infusion), and T2 (one hour after cessation of vasopressin infusion). The Shapiro-Wilk test and stratified distribution plots were used to verify the normality of the distribution of continuous variables. All quantitative data were normally distributed except for the doses of noradrenaline and lactate. Data that were not normally distributed underwent log transformation to achieve close to normal distribution and were then qualified for longitudinal testing.

We categorized patients as responders and non-responders based on changes to sublingual vascular density. Because visible obstructed capillaries do not change TVD but have effects on PVD, we defined responders as those who presented a more than a 10% increase in TVD or PVD after vasopressin infusion. This cut-off was based on a previous study showing that slight changes in the microcirculation are associated with the degree of organ dysfunction 30. Factors associated with the microcirculation response were analyzed using a multivariate regression analysis to develop the predictive model for improvement in microcirculation. Relevant candidate predictor variables at baseline were selected for possible inclusion in the model, including TVD, noradrenaline dose, MAP, HR, CVP, lactate and CI. A backward-elimination approach was tested with all variables, and hypothesis tests were sequentially applied to determine which variables would be removed from the final model. (Predictors were removed if the significance level was more than 0.10.) Discriminative ability was determined with the c-statistic, which is equivalent to the area under the receiver operator characteristic (ROC) curve. The results were then summarized in a graphical assessment of the expected probability for a microcirculatory response based on the final model.

The statistical comparisons at each time point of the study were performed using a generalized mixed-model analysis to estimate the mean response differences and significance of the covariates (global hemodynamic variables and sublingual microcirculation parameters) within the time points (factor) between responders and non-responders (dependent variable). The interaction was tested for each time point to investigate the relationship between changes in hemodynamic variables (MAP, CVP and CI) and microcirculatory parameters. SPSS (version 23.0, SPSS, Chicago, IL, USA) was used for statistical analyses. A *p-*value<0.05 was considered to be statistically significant.

## RESULTS

We screened 116 patients with septic shock who were admitted to the intensive care unit. Fifty-one patients were not included because SDF or the study team were not available. Other reasons for non-eligibility included the following: absence of a Swan-Ganz catheter (n=27), reversal of shock before the baseline assessment (n=10), lack of informed consent (n=3), acute coronary disease (n=2), use of norepinephrine longer than 48 hours (n=2), previous use of vasopressin (n=1), pregnancy (n=1) and death before inclusion (n=1). Eighteen consecutive patients with septic shock were included in the study. Their characteristics at baseline are listed in Table 1. The patients received adrenergic vasopressors for a mean of 27.2±12.2 hours, and the hospital mortality rate was 72%. All patients received 0.04 U/min of vasopressin for one hour, except for one patient who received a dose of 0.02 U/min. At baseline, only four patients received epinephrine (mean dose, 0.28±0.08 mcg/kg/min), and five patients received dobutamine (mean dose, 5.6±2.9 mcg/kg/min). For most patients, we did not change these medication doses; however, for one patient we reduced the epinephrine dose from 0.26 µg/kg/min to 0.13 µg/kg/min instead of reducing the noradrenaline dose because an increase in MAP and excessive tachycardia were evident. In 3 patients, an assessment of the T2 measurement was not possible because there was a clinical indication of tapering of the ventilator parameters or additional sedation.

After vasopressin infusion, the absolute mean reduction in the norepinephrine infusion rate was 32.2±27.4%; *p*=0.001. Nine patients showed improvements in microvascular density after 1 hour of vasopressin infusion. Tables 2 and 3 show the hemodynamic and sublingual microcirculation parameters, respectively, which are stratified by responders and non-responders. Both responders and non-responders had a significant decrease in the noradrenaline dose during the infusion of vasopressin, whereas the noradrenaline dose was significantly higher in the responders than the non-responders both at baseline and during vasopressin infusion (Table 2). Only 5 patients were using dobutamine at baseline; 2 were responders, and 3 were non-responders. After discontinuation of the vasopressin infusion, the noradrenaline dose increased significantly in the non-responder group only, and both responders and non-responders received similar noradrenaline doses at that time. According to our interaction analysis in the generalized mixed-model, the changes in CI, MAP and CVP had no significant effects on TVD or PVD.

Interestingly, the decrease in noradrenaline dosing in the non-responder group was associated with a decrease in CI, oxygen delivery (DO_2_) and oxygen consumption (VO_2_), whereas CI was restored to baseline levels after discontinuation of the vasopressin and the subsequent increase in noradrenaline dosing to maintain MAP (Table 2). The hemodynamic parameters, laboratory variables and microcirculatory measurements in the whole population obtained before and after the vasopressin infusion are listed in the electronic supplementary material (Table S1 and S2).

**Figure 1:**
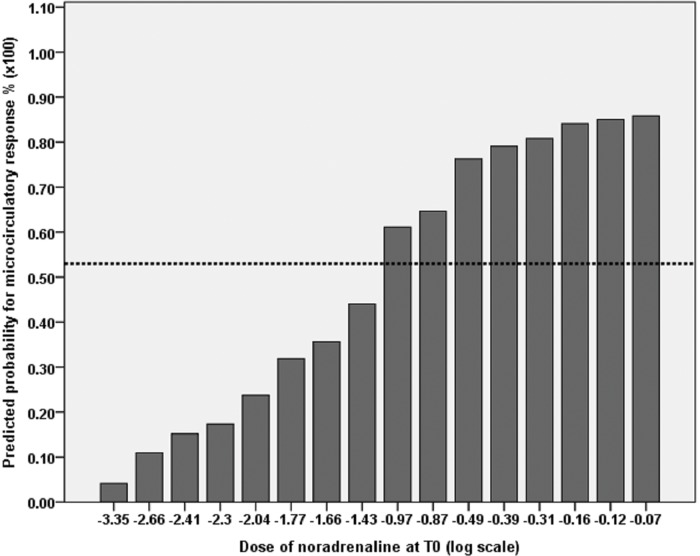
Probability of microcirculatory response based on a dose of noradrenaline at T0 (logarithmic scale). The dotted line shows the threshold value of the predicted probability of 53% for patients receiving a noradrenaline dose higher than 0.38 mcg/kg/min (78% sensitivity and 77% specificity).

In a multivariate regression model (Table S3), the strongest predictor for an improvement in the microcirculation was the baseline dose of noradrenaline (OR=4.5; 95% CI: 1.2-17.0; *p*=0.027). Figure 1 shows the probability of a microcirculatory response based on the noradrenaline dose. The model showed adequate calibration, good discrimination and an area under the ROC curve of 0.85 (95% CI: 0.66-0.99). The probability for 78% sensitivity is indicated, which corresponds to a predicted probability of more than 50% for patients receiving a noradrenaline dose higher than 0.38 mcg/kg/min (log scale >-0.97 in the graph). Table 4 shows the final model with predicted probabilities for a microcirculatory response to vasopressin according to different infusion doses of noradrenaline to facilitate practical application.

## DISCUSSION

The primary finding of this study was that the improvement in the microcirculatory parameters after vasopressin infusion in the septic shock patients was highly associated with the level of noradrenaline dependency and was independent of systemic hemodynamic parameters (CI, MAP, and CVP). This finding suggests that the patient’s noradrenaline dose is directly proportional to the likelihood that the patient will respond to vasopressin infusion. For clinical decision making, patients receiving a noradrenaline dose above 0.38 mcg/kg/min (independent of hemodynamic conditions) are candidates for vasopressin use with a 53% probability of recruiting the microcirculation. These findings, which were not previously reported, may help with selecting patients who may benefit from the association of vasopressin according to their noradrenaline dependency status.

The effects of vasopressin in the microcirculation have already been demonstrated by Morelli’s group, which showed that continuous infusions of low-dose vasopressin and terlipressin improved the MFI after 6 hours. However, the authors found similar results in the control group, which suggests that these changes may not have been related to vasopressin use 31. In our study, we assessed only the short-term effects of vasopressin. Although a subgroup analysis of the VASST study has suggested that patients with less severe septic shock, who require 5 to 14 mcg of noradrenaline per minute, would have better clinical outcomes with the use of vasopressin 32 at the microcirculatory level; however, our results point in the opposite direction. Our multivariate model showed that the most clinically useful predictor for a microcirculatory response was the noradrenaline dose required to maintain a MAP above 65 mmHg. Vasopressin infusion also reduced norepinephrine requirements without any significant adverse effect during this short period of infusion.

Microcirculatory alterations can be observed even when systemic hemodynamics are within satisfactory goals. The independence of the microcirculation parameters has been previously reported 18 33 34,35. However, microcirculatory perfusion can be affected by cardiac output and arterial pressure when these variables are critically altered. We found that non-responders had a significant reduction in their cardiac output, DO_2_ and VO_2_ during vasopressin infusion, which suggests that changes in the systemic hemodynamics may lead to microcirculatory alterations. However, the reduction in DO_2_ was not followed by an increase in arterial lactate or differences in other tissue perfusion parameters, suggesting that these macrohemodynamic alterations were not clinically relevant. However, changes in the CI were not associated with improvement in the microcirculation in our multivariate model. This result highlights the relevance of measuring CI in studies aiming to assess microcirculation even if the enrollment rate is compromised. In fact, one of the major reasons for not enrolling patients in our study was the absence of CI measurements.

Additionally, the reduction in CI may have been related to the reduction in the noradrenaline dose and, consequently, to its inotropic and chronotropic effects or its effect on venous return. A non-significant reduction in the SI was evident during vasopressin infusion. The reduction in noradrenaline dose might be considered a beneficial effect as increasing evidence indicates that the excessive use of catecholamines is associated with potential iatrogenic complications. These potential harmful effects are not exclusively related to well-known hemodynamic effects, namely, increased energy expenditure; excessive vasoconstriction; and splanchnic hypoperfusion with altered gut motility, function and potential bacterial translocation. Convincing evidence exists showing that the phagocytic capacity of macrophages and neutrophils is inhibited 36, together with lymphopenia and a shift toward a Th2 pattern 37. Bacterial virulence and proliferation is also enhanced 7. Metabolic changes include hyperglycemia, hypertriglyceridemia and thyroid hormone alterations. Coagulation disorders with enhanced clot formation and a reduction in fibrinolysis have also been reported 7. Thus, the use of vasopressin is part of the decatecholaminization strategy that has gained increased support in critical care.

Although our study was not designed to assess causality, we can provide several hypotheses. Vasopressin led to a reduction in the noradrenaline dose in all patients. This result suggests that the use of receptors other than adrenergic receptors will lead to a better response in vasopressor tone and, consequently, to a reduction in the need for vasopressors. Reducing the excessive vasopressor effect of noradrenaline may have led to an increase in microcirculatory density and perfusion in the responders. Non-responders were using a smaller dose of noradrenaline, which suggests less severe disease and vasodilation. In this context, vasopressin might have caused excessive vasoconstriction, as suggested by the finding of a decrease in PVD. Moreover, the potential negative effects of vasopressin in cardiac output may have played a role. Patients who had a smaller reduction in CI after vasopressin infusion would be likely to show improvements in microcirculation. The differences between the responders and non-responders regarding the impact of vasopressin on CI, DO_2_ and VO_2_ were interesting. For the non-responders, the CI reduction did not compromise the microcirculation but was associated with a decrease in VO_2_ that was reversed when vasopressin was suspended. This finding hypothetically suggests that if vasopressin is used, a decrease in noradrenaline requirements may occur if the microcirculation does not improve the overall effect of vasopressin, which might be harmful. However, these notions are speculations because distinguishing the effects of vasopressin on contractility from its vasoconstrictive effects on microcirculation is difficult.

Although this study provides novel observations, a fixed dose of vasopressin was used, which could be considered insufficient to show any beneficial or harmful effects. A previous study suggested that a higher dose of vasopressin (0.067 U/min) would restore hemodynamics more effectively and that the dose was not associated with a high incidence of adverse effects 38. Another limitation of the present study is the absence of a control group. However, this study was designed to allow every patient to serve as his or her own control, which minimizes bias. Therefore, the significant improvement or decrease in the sublingual microcirculation parameters during and after vasopressin infusion strengthens our findings. An additional limitation is the short observation period of our study as a longer AVP infusion period might have a different impact on the microcirculatory response. Notably, the high severity of illness in our population is another limitation worth considering. These patients had a mean organ dysfunction incidence of 4.2 and a high mortality rate. Thus, the effects of vasopressin may be different in patients with a less severe shock.

In conclusion, the clinical monitoring of the sublingual microcirculation can help identify patients with septic shock that might benefit from the association of vasopressin. Our results suggest that a noradrenaline dose above 0.38 mcg/kg/min might be a good predictor for the microcirculatory response to the vasopressin infusion. Additional research that explores different microcirculatory beds and uses different measurement tools for assessing microcirculation will improve our knowledge concerning the role of vasopressin in septic shock resuscitation.

## AUTHOR CONTRIBUTIONS

Nascente AP and Machado FR are the guarantors of the entire manuscript. Nascente AP, Machado FR and Freitas FG designed the study. Nascente AP collected all of the data. All authors helped with the data interpretation and drafting of the manuscript. All authors revised and approved the final version of the manuscript.

## Figures and Tables

**Table 1:** Demographic data and sepsis characteristics.

Variable	All patients (n=18)	Responders (n=9)	Non-responders (n=9)	*p* value
Age (years)	62.3±17.8	63.9±17.4	60.2±19.8	0.601
Males	11 (61.1)	6 (66.7)	5 (55.6)	0.629
APACHE II	13.4±4.6	14.1±6.1	12.9±3.1	0.630
Admission SOFA	11 (8, 13)	10 (7, 12.5)	11 (9.5,13)	
Admission category				0.881
Ward	7 (38.9)	4 (44.4)	3 (33.3)	
Surgery	9 (50)	4 (44.4)	5 (55.6)	
Emergency department	2 (11.1)	1 (11.1)	1 (11.1)	
Patient category				0.774
Medical	9 (50)	5 (55.6)	4 (44.4)	
Elective surgical	3 (16.7)	2 (22.2)	1 (11.1)	
Emergency surgical	6 (33.4)	2 (22.2)	4 (44.4)	
Type of infection				0.287
Community	5 (27.8)	1 (11.1)	4 (44.4)	
Nosocomial - ward	8 (44.4)	5 (55.6)	3 (33.3)	
Nosocomial - ICU	5 (27.8)	3 (33.3)	2 (22.2)	
Site of infection				0.164
Lung	5 (27.8)	2 (22.2)	3 (33.3)	
Abdominal	6 (33.3)	2 (22.2)	4 (44.4)	
Urinary	2 (11.1)	0 (0)	2 (22.2)	
Catheter	1 (5.6)	1 (11.1)	0 (0)	
Bloodstream	2 (11.1)	2 (22.2)	0 (0)	
Unknown	2 (11.1)	2 (22.2)	0 (0)	
Organ dysfunctions (number)	4.17±1.61	3.78 ±1.4	4.56±1.8	0.322
Duration of organ dysfunction (hours)	36.54 (30.0, 51.8)	39.9 (29.7, 56.5)	34.7 (27.7, 45.4)	0.606
Duration of vasopressor use (hours)	27.2±12.2	24.7±13.8	29.3±10.9	0.457
ICU mortality	11 (61.1)	6 (66.7)	5 (55.6)	0.629
Hospital mortality	13 (72.2)	6 (66.7)	7 (77.8)	0.599

APACHE, Acute Physiological Chronic Health Evaluation; SOFA, Sequential Organ Failure Assessment; ICU, intensive care unit. The results are expressed as the number (%), mean±standard deviation or median (25%, 75%).

**Table 2:** Global hemodynamic parameters and noradrenaline dose recorded at the three different time points during the study protocol and categorized according to responders and non-responders.

Variables	Subgroup	T0 (n=18)	T1 (n=18)	T2 (n=15)
Noradrenaline (µg/K/min)	Non-responder	0.28 (0.17-0.41)	0.15 (0.09-0.24)*	0.39 (0.23-0.56)*
	Responder	0.68 (0.40-1.04)	0.49 (0.38-0.88)*	0.59 (0.42-0.88)
	*p* value	0.008	0.010	0.320
HR (bpm)	Non-responder	101 (12)	96 (14)*	101 (11)*
	Responder	113 (25)	109 (23)	116 (20)*
	*p* value	0.220	0.150	0.060
MAP (mmHg)	Non-responder	74.6 (8)	74.4 (5)	70.1 (5)
	Responder	70.4 (3)	70.8 (3)	70.5 (4)
	*p* value	0.152	0.089	0.370
CI (L/min.m^2^)	Non-responder	4.5 (1.28)	3.6 (1.04)*	4.0 (0.96)*
	Responder	4.2 (1.20)	3.7 (0.91)	3.9 (0.82)
	*p* value	0.560	0.900	0.510
SI (mL/m^2^)	Non-responder	45.2 (15.2)	38.8 (12.9)	40.5 (11.5)
	Responder	36.4 (7.1)	34.6 (7.2)	34.7 (7.9)
	*p* value	0.146	0.405	0.262
Lactate (mg/dL)	Non-responder	21.3 (14.0-32.0)	18.9 (12.9-27.1)	25.0 (12.9-43.8)
	Responder	30.8 (24.0-43.8)	28.7 (21.9-46.0)	27.1 (23.1-32.1)
	*p* value	0.22	0.15	0.23
SvO_2_ (%)	Non-responder	71.4 (6.9)	69.1 (9.5)	73.3 (8.8)
	Responder	75.7 (5.7)	72.5 (5.4)	71.7 (5.4)*
	*p* value	0.170	0.360	0.450
a-vCO_2_	Non-responder	3.0 (1.3)	4.8 (1.6)	4.6 (2.0)
	Responder	4.1 (2.2)	5.5 (3.9)	4.8 (1.3)
	*p* value	0.28	0.44	0.85
DO_2_ (mL/min)	Non-responder	791 (673-1435)	739 (568-986)*	731 (542-1231)
	Responder	836 (678-1080)	738 (613-955)	740 (673-1116)
	*p* value	0.51	0.89	0.70
VO_2_ (mL/min)	Non-responder	188 (152-370)	171 (132-292)*	188 (156-278)*
	Responder	197 (145-243)	199 (145-206)	196 (173-221)
	*p* value	0.19	0.42	0.29
Extraction rate (%)	Non-responder	25.6 (21-26)	23.1 (19-34)	23.3 (21-35)
	Responder	22.0 (18-31)	22.6 (18-28)	23.7 (17-29)
	*p* value	0.49	0.29	0.39

Time points are defined as before (T0), during (T1) and after (T2) vasopressin infusion. There were 9 responders and 9 non-responders. HR, heart rate; MAP, mean arterial pressure; CI, cardiac index; SI, systolic index; SvO_2_, oxygen mixed venous saturation. Data are expressed as the mean (standard deviation) or median (25%, 75%).

* *p*<0.05 *vs*. previous time point within the same group.

**Table 3:** Sublingual microcirculation parameters recorded at the three different time points during the study protocol and categorized according to responders and non-responders.

Variable	Subgroup	T0	T1	T2
TVD (mm/mm^2^)	Non-responder	15.9 (2.5)	14.4 (1.8)*	14.8 (0.58)
	Responder	14.3 (1.7)	16.3 (1.6)*	15.2 (1.57)*
	*p* value	0.13	0.03	0.35
PVD (mm/mm^2^)	Non-responder	13.9 (2.7)	12.4 (1.8)*	12.6 (1.3)
	Responder	12.1 (1.9)	14.4 (1.4)*	13.0 (1.9)*
	*p* value	0.11	0.02	0.47
PPV (%)	Non-responder	87.3 (7.6)	86.5 (6.4)	85.0 (7.8)
	Responder	83.5 (6.0)	89.0 (4.8)*	86.6 (7.0)
	*p* value	0.26	0.36	0.62
MFI	Non-responder	2.7 (0.2)	2.8 (0.2)	2.7 (0.2)
	Responder	2.7 (0.1)	2.6 (0.3)	2.7 (0.2)
	*p* value	0.69	0.15	0.89

Time points are defined as before (T0), during (T1) and after (T2) vasopressin infusion. There were 9 responders and 9 non-responders. TVD, total vascular density; PPV, proportion of perfused vessels; PVD, perfused vascular density; MFI, microcirculatory flow index; Data are expressed as the mean (standard deviation) or median (25%, 75%).

* *p*<0.05 *vs*. previous time points within the same group.

**Table 4:** Predicted probability of the different doses of noradrenaline for microcirculatory responses with their corresponding sensitivity and specificity.

Noradrenaline dose (mcg/kg/min)	Predicted probability for the microcirculatory response to vasopressin	Sensitivity*	Specificity*
**>0.17**	27.8%	89%	56%
**>0.38**	53.0%	78%	77%
**>0.68**	77.6%	67%	99%

* ROC curve, AUC = 85%, *p*=0.013.

**Table S1:** Changes in hemodynamic, respiratory and metabolic variables after vasopressin infusion – global analysis.

Variables	T0 (n=18)	T1 (n=18)	T2 (n=15)
Norepinephrine (μg/kg/min)	0.41 (0.23, 0.90)	0.31 (0.09, 0.76)*	0.56 (0.23, 0.85)*
Heart rate (bpm)	107.4±19.7	102.7±19.9*	110.53±18.9*
MAP (mmHg)	72.56±6.2	72.7±4.4	70.4±4,0
CVP (mmHg)	11.6±5.0	11.8±4.6	11.5±4.5
PAOP (mmHg)	9.6±4.5	10.6±3.7	9.0±4.2
mPAP (mmHg)	27.6±8.0	26.9±7.4	26.7±7.5
CO (L/min)	7.8±2.9	6.7±2.4*	7.1±2.2*
CI (L/min.m^2^)	4.4±1.2	3.7±0.9*	4.0±0.8*
SI (ml/beat/m^2^)	40.8±12.4	36.9±10.6	37.0±9.6
PPV (%)	5.0 (3.0, 10.5)	4.0 (2.1, 8.7)	4.7 (4.0, 5.7)
DO_2_ (ml/min)	813.5 (676.2, 1153.0)	738.5 (595.5, 939.5)*	740.0 (643.0, 1048.0)
VO_2_ (mL/min)	188.0 (142.0, 246.0)	171.0 (140.0, 203.0)*	196.0 (164.0, 225.0)*
Extraction rate (%)	24.6±7.1	25.6±7.9	25.0±7.1
PEEP (cmH_2_O)	9.6±3.6	9.6±3.6	9.5±3.9
FiO_2_ (%)	0.40 (0.40, 0.50)	0.40 (0.40, 0.50)	0.4 (0.4, 0.5)
pO_2_/FiO_2_ ratio	231.2 (169.4, 263.5)	222.5 (187.6, 255.7)	203.0 (185.0, 231.0)
Lactate (mg/dL)	30.3±17.3	27.6±15.8	29.6±15.4
a-vCO_2_ (mmHg)	3.7 (2.5, 4.7)	4.6 (3.2, 6.5)	4.7 (4.0, 5.7)
Urine output (ml/kg/h)	0.2 (0.0, 1.4)	0.1 (0.0, 0.4)	0.0 (0.0, 0.5)
SvO_2_ (%)	73.6±6.5	70.9±7.7	72.4±6.7
Haemoglobin (g/dL)	9.0±1.4	8.8±1.4	8.8±1.5

Time points are defined as before (T0), during (T1) and after (T2) vasopressin infusion. MAP, mean arterial pressure; CVP, central venous pressure; PAOP, pulmonary artery occluded pressure; PAP, pulmonary artery pressure; CO, cardiac output; CI, cardiac index; SI, systolic index; PPV, pulse pressure variation; DO_2_, oxygen delivery; PEEP, positive end expiratory pressure; FiO_2_, fraction of inspired oxygen; pO_2_, oxygen partial pressure; ΔCO_2_, venous-arterial CO_2_ gradient; SvO_2_, oxygen mixed venous saturation. Data are expressed as number (%), mean±standard deviation or median (25%, 75%).

* *p*<0.05 *vs*. previous time point.

**Table S2:** Changes in the microcirculatory variables after vasopressin infusion – global analysis.

Variable	T0	T1	T2
TVD (mm/mm^2^)	15.1± 2.2	15.2±1.8	15.2±1.5
PVD (mm/mm^2^)	13.0±2.4	13.4±1.8	12.9±1.7
PPV (%)	87.1 (83.0, 90.5)	88.6 (84.1, 90.5)	85.8 (83.4, 90.5)
MFI	2.7 (2.6, 2.9)	2.9 (2.5, 3.0)	2.7 (2.5, 3.0)

Time points are defined as before (T0), during (T1) and after (T2) vasopressin infusion. TVD, total vascular density; PPV, proportion of perfused vessels; PVD, perfused vascular density; MFI, microcirculatory flow index; HI, heterogeneity index. Data are expressed as mean±standard deviation or median (25%, 75%).

* *p*<0.05 *vs*. previous time point.

**Table S3:** Multivariable regression model to changes in microcirculatory response.

Independent variable	β coefficient±SE	OR 95% CI	*p*-value
TVD	-0.73±0.4	0.479 (0.180 – 1.272)	0.14
HR	0.013±0.07	1.013 (0.877 – 1.172)	0.85
MAP	-0.15±0.31	0.855 (0.463 – 1.579)	0.61
CVP	0.09±0.34	1.104 (0.566 – 2.154)	0.77
Lactate	-0.07±0.068	0.925 (0.811 – 1.056)	0.25
CI	-0.83±0.81	0.433 (0.088 – 2.128)	0.30
Noradrenaline dose*	4.8±2.3	5.611 (1.130 – 27.861)	0.03

* Noradrenaline dose was used as a categorized variable.

Backward-elimination approach was tested and predictors were removed if significance level were more than *0.10*. Independent variables at baseline were included: Total Vascular Density (TVD), hear rate (HR), mean arterial pressure (MAP), central venous pressure (CVP), lactate, cardiac index (CI) and noradrenaline dose.
